# Evaluation of *Bacillus paramycoides* Strains Isolated from *Channa* Fish sp. on Growth Performance of *Labeo rohita* Fingerlings Challenged by Fish Pathogen *Aeromonas hydrophila* MTCC 12301

**DOI:** 10.3390/microorganisms11040842

**Published:** 2023-03-25

**Authors:** Sufiara Yousuf, Mamdoh T. Jamal, Radwan Kahalid Al-Farawati, Bandar Ahmad Al-Mur, Rahul Singh

**Affiliations:** 1 Department of Zoology, School of Bioengineering and Biosciences, Lovely Professional University, Phagwara 144411, Punjab, India; 2 Department of Marine Biology, Faculty of Marine Sciences, King Abdul-Aziz University, Jeddah 21589, Saudi Arabia; 3 Department of Marine Chemistry, Faculty of Marine Sciences, King Abdul-Aziz University, Jeddah 21589, Saudi Arabia; 4 Department of Environmental Sciences, King Abdul-Aziz University, Jeddah 21589, Saudi Arabia

**Keywords:** *Bacillus paramycoides*, *Labeo rohita*, *Aeromonas hydrophila*, antimicrobial activity

## Abstract

Probiotics play vital roles in improving growth, survival, and immune responses and inhibit the growth of pathogenic bacteria in freshwater fish. This study was conducted to isolate potential probiotics from *Channa punctatus* and *Channa striatus* and to evaluate their effect on *Labeo rohita* fingerlings. Among the isolates, *Bacillus paramycoides* PBG9D and BCS10 (1) exhibited antimicrobial activity against the fish pathogen *Aeromonas hydrophila*. Both strains showed tolerance to acidic and alkaline pH (2, 3, 4, 7, and 9) and bile salts (0.3%) and exhibited strong adhesion capacity. After in-vitro assessment, these strains were evaluated on the growth performances of rohu fingerlings challenged by *Aeromonas hydrophila* for 4 weeks. The study consisted of six groups, each containing 6 fish. Group (I) was the control, fed a basal diet; group (II) contained a pathogen and was also fed a basal diet; group (III & IV) was given a probiotic supplemented experimental diet; Fourth group (V & VI) contained a pathogen and was given a probiotic supplemented experimental diet. After the 12th day of experiment, rohu fingerlings of pathogen (II) and probiotic + pathogen (V & VI) groups were intraperitoneally injected with 0.1 mL of *Aeromonas hydrophila*. After 4 weeks, no significant differences in weight gain, weight gain %, and feed conversion ratio were observed in probiotic (III & IV)- fed groups compared to control. However, the specific growth rate was significantly improved in probiotic fed groups compared to other groups. Survival rate and condition factor were significantly similar in all groups. After injection, abnormal swimming, loss of appetite and weight loss were observed in the pathogen (II) group, while no such symptoms were found in the probiotic + pathogen (V & VI)- groups, confirming the effects of probiotics. The overall results of the study revealed that dietary supplementation with *Bacillus paramycoides* strains could improve the specific growth rate and disease resistance against *Aeromonas hydrophila* in *Labeo rohita*.

## 1. Introduction

Globally, carp production contributes more than 72% of total freshwater aquaculture production, among which, *Labeo rohita* accounts for more than 15%, with 2 million tonnes of production in 2018 [1,2]. However, in India, rohu accounts for about 35% of major carp production. Owing to the increasing consumer demand, fast growth rate and the ability to thrive in a range of agro-climatic conditions, *Labeo rohita* is deemed as one of the most preferred carp for aquaculture among Indian major carps [3]. In terms of nutrition, rohu is a good source of protein and n-3 PUFAs, with the highest protein content among major carp species [4]. The main producer countries of this species include India, Nepal, Bangladesh, Myanmar, Laos, Thailand, and some African countries [5]. However, the serious problem that affects production of this species arises from high stocking densities for commercial purposes. This creates stressful conditions for the fish, leading to infectious disease outbreaks, which result in economic losses [6]. Several pathogenic micro-organisms responsible for development of infectious diseases belong to the following genera: *Aeromonas*, which causes motile *Aeromonas* septicaemia; *Pseudomonas*, which causes haemorrhagic septicaemia; *Streptococcus*, which causes streptococcosis; Vibrio, which causes vibriosis; and *Edwardsiella*, which causes Edwardsiellosis [7,8,9,10].

To combat infectious diseases, antibiotics and other chemotherapeutic drugs have been used for many years, which results in the development of drug resistant pathogens, affects the host’s beneficial gut bacteria, and causes toxicity in consumers that can lead to death. For example, chloramphenicol residues increase the risk of cancer, and even extremely low amounts can cause aplastic anemia, a disorder in which the bone marrow stops generating red and white blood cells and is often deadly. This drug inhibits protein synthesis in mitochondrial ribosomes and deteriorates their morphology and functioning [11]. Other antibiotics, such as sulphamethazine, oxytetracycline, and furazolidone, have immunopathological and carcinogenic effects [12]; gentamicin has mutagenic and nephropathic effects; and penicillin causes allergy [13]. Hence, it is important to adopt safe and eco-friendly alternatives, i.e., the use of probiotics.

Probiotics are live bacteria that, when delivered in adequate proportions, provide health benefits to the host and environment without any side effects [14]. Moreover, probiotics provide their beneficial effects through a variety of routes, which include an enhanced epithelial barrier, increased adhesion to the intestinal wall, production of antimicrobial substances, and immune modulation [15,16]. Additionally, probiotics positively influence nutrition, feed utilization, gut morphology, and the functioning of the host [17,18]. In the environment, probiotics act in multiple ways that include increasing beneficial bacterial populations, inhibiting the growth of harmful algae (blue-green cyanobacteria) and pathogenic bacteria, and increasing nutrient and oxygen concentrations [19,20]. *Lactobacillus* spp., *Bacillus* spp., *Saccharomyces cerevisiae*, and *Lactococcus* spp. have all been administered as probiotics in aquaculture to enhance carp growth [21,22]. However, given the efficacy of probiotics, the scientific community continues to search for novel strains that will result in maximum production and higher benefits for farmers.

For sustainable development, the scientific community has historically focused on isolating novel probiotic strains from terrestrial sources, such as dairy products, fermented foods, and livestock. However, novel probiotics derived from aquatic sources can work better in the original habitat, result in better colonization, and have the ability to restore homeostatic conditions [23,24]. *Bacillus* sp. isolated from the gastrointestinal tract of healthy *Labeo rohita* provide protection against *Aeromonas hydrophila* infection by improving immune responses in rohu [25]. *Lactobacillus fermentum* URLP18 isolated from freshwater fish possesses high probiotic potential, and the results suggest that the isolate would be a promising candidate for freshwater aquaculture [26]. Furthermore, *Bacillus subtilis* MBTDCMFRI Ba37 isolated from a sediment sample improved survival rate, growth, and immune parameters in juvenile *Etroplus suratensis*. The results of that study demonstrated that the isolate could be effective in managing diseases in aquaculture and improving the host’s health [27].

Wild fish possess a more diverse gut microbiome than farmed fish due to variability in nutrition supplies and other factors in the ecosystem [28]. *Channa striatus* and *Channa punctatus* are snake-headed fish species, commonly known as mudfish, and belong to the Channidae family. These species can be found in inland water bodies, freshwater plains, muddy lake bottoms, canals, and swamps. Isolating bacteria from fish of different orders, bottom dwellers, and fish exposed to biofilms provides the possibility of isolating a novel strain with probiotic potential. Therefore, looking into new probiotic strains with antibacterial properties can help to protect fish from infection. Hence, from the above context, the aim of our study was to isolate and identify probiotic strains from the intestinal tracts of *Channa punctatus* and *Channa striatus* and evaluate their potential in rohu fingerlings using a range of in vitro tests with in vivo safety assessment.

## 2. Materials and Methods

### 2.1. Sample Collection

**Figure 1:**
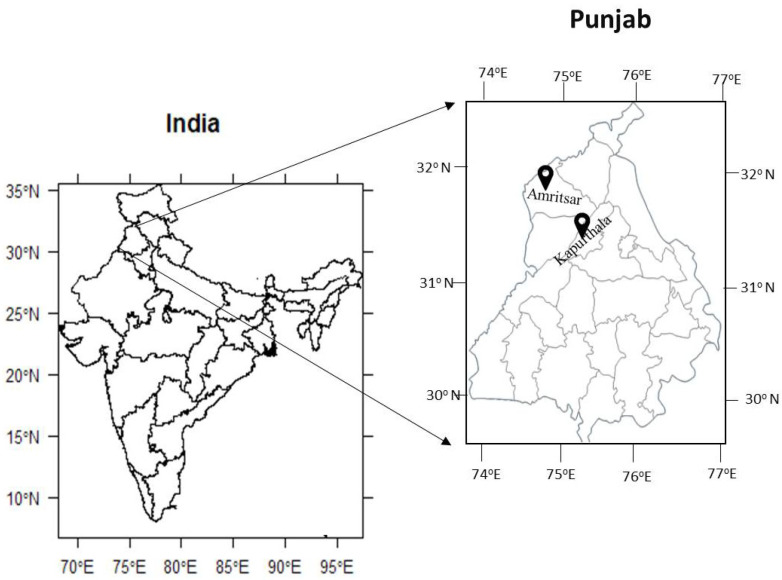
Map representing the sample collection sites of Majha and Doaba regions in Punjab, India.

Samples of *Channa Punctatus* and *Channa striatus* were collected from the Doaba and Majha regions of Punjab, India. The Doaba region lies between the Beas and Satluj rivers of Punjab, while the Majha region is the land between the Beas and Ravi rivers, also known as the heartland of Punjab, as illustrated in Figure 1. Fish were transported to the laboratory in sterile bags under proper maintenance to conduct further studies.

### 2.2. Isolation of Gut Bacteria

In this study, the surface of each fish sample was cleaned with 70% ethanol to prevent bacterial contamination. Under aseptic conditions, intestines were removed, dissected, and washed with saline water to eliminate waste materials, and then homogenized in saline water. The homogenate was streaked on MRS (De Man, Rogosa and Sharpe agar) and Bacillus differentiation agar (BDA)- (HiMedia- Mumbai, India) containing petri plates, followed by incubation at 37 °C for 24 h. Morphologically different colonies were picked up with a loop and streaked onto fresh MRS and BDA plates to obtain pure culture. Colonies were differentiated on the basis of shape (round, irregular, and rhizoid), elevation (flat, knobby, raised, and cushion-shaped), and pigmentation (color). The pure culture was stored in nutrient broth and supplemented with 40% glycerol at −80 °C.

### 2.3. Evaluation of Antimicrobial Activity

The antimicrobial activity of the isolates was evaluated, using the cross-streak method [29], against the pathogens *Aeromonas hydrophila* (MTCC 12301) and *Pseudomonas aeruginosa* (MTCC 7453). *Aeromonas hydrophila* was cultured on Luria agar, while *Pseudomonas aeruginosa* was cultured on nutrient agar at 37 °C for 24 h. *Aeromonas hydrophila* is the most common fish pathogen causing haemorrhagic septicaemia in stressed fish [30]. However, *Pseudomonas aeruginosa* causes haemorrhagic septicaemia, gill necrosis, and abdominal distension in fish under stressful conditions [9]. In brief, Mueller–Hinton agar plates were prepared. The isolated strains were streaked across the diameter of the plate, while the pathogen strain was streaked along the perpendicular of the plate, followed by incubation at 37 °C for 24 h. After incubation, inhibition was determined by zone of clearance (mm) at intersection points rated as +++ for complete inhibition, ++ for moderate inhibition, and + for low inhibition.

### 2.4. pH and Bile Tolerance

For the determination of pH tolerance of the isolates {PBG9D and BCS10 (1)}, loops of freshly cultured strains were inoculated into nutrient broths with varying pH (2, 3, 4, 7, and 9). The pH was adjusted using 1 N HCl and 1N NaOH. The inoculated tubes were incubated at 37 °C for 24 h. After 24 h, the optical density (OD) was measured using a spectrophotometer at 600 nm and compared with uninoculated broths as controls [31]. The intestinal fluid pH of *Labeo rohita* ranges from 6.8–7. In the bile salt tolerance test, the bacterial isolates were inoculated into nutrient broth (HiMedia) containing 0.3% bile salt and incubated at 37 °C for 24 h. After incubation, absorbance was measured at 600 nm using a spectrophotometer [32]. Nutrient broth without bile salt was used as a control.

### 2.5. Antibiotic Susceptibility Test

Antibiotic susceptibility of bacterial isolates was evaluated by the disk diffusion method against seven antibiotics, which included ampicillin (25 µg), amoxicillin (10 µg), kanamycin (30 µg), neomycin (30 µg), erythromycin (15 µg), penicillin (10U), and tetracycline (30 µg). In brief, 100mL of bacterial isolates were spread on Mueller–Hinton agar plates (Hi-media). Antibiotic discs were placed on the surfaces of the plates and incubated at 37 °C for 24 h. After incubation, the zone of inhibition was measured in mm [1,26,33].

### 2.6. Adhesion and Biofilm Formation

For quantitative analysis of biofilm formation and adhesion properties of the bacterial isolates, the standard tube method was followed. In brief, bacterial isolates {PBG9D and BCS10 (1)} were inoculated in test tubes containing 5 mL of Luria–Bertani broth (HiMedia) and incubated for 24 h at 37 °C without shaking. After that, the test tubes containing cultures were poured out and rinsed meticulously with water, fixed with 2.5% glutaraldehyde, again washed with water, and then stained with crystal violet solution (0.4%) (HiMedia). After crystal violet dissolution with ethanol–acetone (80:20 *vol*/*vol*) the absorbance was measured at 600 nm [34].

### 2.7. Identification of Bacterial Isolates

Bacterial isolates were characterized using biochemical tests including Gram staining, the catalase test, the motility test, the gelatin test, the indole test, the urease test, H2S production, the lactose and glucose fermentation test, and the methyl red and Voges–Proskeur test, as mentioned in Bergey’s Manual of Systematic Bacteriology [35].

For molecular characterization, 16s ribosomal RNA gene amplification was conducted at the Microbial Type Culture Collection (MTCC), Chandigarh, Punjab, India. The obtained nucleotide sequences were compared with the sequence of *Bacillus* sp. in the National Center for Biotechnology Information (NCBI) using the BLAST program. The gene sequences of PBG9D and BCS10 (1) were submitted to NCBI and accession numbers were obtained (PBG9D- OQ300496, BCS10 (1)-OQ303631). Evolutionary analysis was conducted in MEGA7.

### 2.8. In Vivo Setup

The experiment was conducted during May–June 2022 for 4 weeks in the Zoology Laboratory, School of Bioengineering and Biosciences, Lovely Professional University, Punjab, India. The laboratory had an ambient temperature of 28–30 °C and was well ventilated. Tanks with a capacity of 40 litres (L) were used for the experiment, and continuous aeration was provided using an air pump (RS-9801). The tanks were maintained under a natural light/dark photoperiod.

#### 2.8.1. Preparation of Probiotic Feed

The commercial fish feed ‘Hopar Grow’ (made in Thailand) was utilized as a basal diet with nutrient content of 30% crude protein, 3% crude fat, 4% crude fibre, 10% crude ash, and 10% moisture during the entire trial period. The probiotic-enriched diet was prepared per the procedure followed by Ramesh et al. and Adorian et al. [1,36]. Briefly, the pure bacterial culture was inoculated into nutrient broth and kept in an incubator at 37 °C for 24 h. The broth culture was centrifuged at 3000× *g* for 10 min to form a pellet. The pellet was washed three times with phosphate-buffered saline (PBS, pH 7.2) and then resuspended in PBS. Bacterial concentration was standardized by UV-Vis spectrophotometer. At optical density (OD 600), the absorbance was set to 0.3 to standardize the no. of bacteria (10^8^ CFU/mL) [37,38]. Probiotic-enriched diets were prepared by spraying the bacterial broth onto the basal diet and mixing meticulously to achieve a dose of 1 × 10^8^ CFU/g. The experimental diets were air-dried in laminar airflow using a blower until the moisture levels were below 10%. Then, these feeds were labeled accordingly and stored in airtight containers at 4 °C until use. The diets were prepared weekly to ensure the viability of the probiotics [39]. The basal diet sprayed with only sterile PBS was used as a control feed.

#### 2.8.2. Fish and Experimental Design

**Figure 2:**
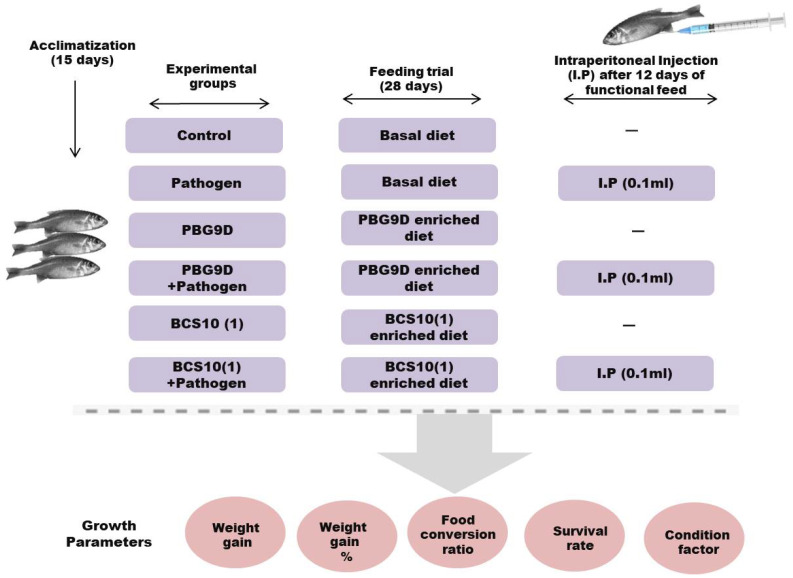
Schematic diagram representing experimental design for the evaluation of probiotic strains against *Aeromonas hydrophila*.

Rohu fish (*Labeo rohita*) fingerlings with an average weight of 5–12 g were obtained from a commercial fish farm located in Khanna City, Ludhiana District of Punjab. The fish were acclimatized for 15 days and were fed with commercial feed at a rate of 3% of body weight. Fish of size 9.31 ± 1.21 cm were divided into 6 tanks, with 6 fish in each group. The water in the tanks was changed every three days, and water quality (pH 6.9–7.4) was examined daily throughout the experiment. The experiment was conducted in six groups, including control (I), pathogen (II) (*Aeromonas hydrophila*), probiotic (III) (PBG9D), probiotic (IV) (BCS10 (1)), probiotic with pathogen (V) (PGB9D + *Aeromonas hydrophila*), and probiotic with pathogen (VI) (BCS10 (1) + *Aeromonas hydrophila*) (Figure 2). Fish in the control (I) and pathogen (II) groups were supplemented with a basal diet, while fish in the probiotic (III) and (IV) and probiotic with pathogen (V) and (VI) groups were fed with the probiotic-enriched feed with a dose of 1 × 10^8^ CFU/g. The feed rate was 3% of body weight one time per day at 4:00 pm for 4 weeks.

#### 2.8.3. Challenge Test

After 12 days of functional feed, fish were challenged intraperitoneally with 0.1 mL of *Aeromonas hydrophila* (10^8^ CFU/mL) [40,41]. *Aeromonas hydrophila* is a common fish pathogen that causes haemorrhagic septicaemia and ulcerative syndrome in fish [42,43]. Fish were anesthetized by adding clove oil (2 drops) into the water for 2–3 min and then were injected with the test pathogens. Fish in the pathogen (II) and probiotic with pathogen (V) and (VI) groups were immunized and were observed daily for 16 days. Mortality, swimming abnormalities, and signs of infection were recorded on a daily basis.

#### 2.8.4. Growth Performances

At regular intervals, all fish were measured and weighed individually for data calculation of growth parameters. Growth performance factors, such as weight gain, weight gain %, specific growth rate %, survival rate (%), feed conversion ratio, and condition were calculated according to the following formulas [36].

Weight gain (WG) = Final weight − Initial weight

$$\mathrm{Weight}\;\mathrm{gain}\;\%\;\left(\mathrm{WG}\right)=100\frac{\left(\mathrm{Final}\;\mathrm{weight}\;-\;\mathrm{Initial}\;\mathrm{weight}\right)}{\mathrm{Initial}\;\mathrm{weight}}$$

$$\mathrm{Specific}\;\mathrm{growth}\;\mathrm{rate}\;\%\;\left(\mathrm{SGR}\right)=100\frac{\left({\mathrm{lnW}}_{f}{-\;\mathrm{lnW}}_{i}\right)}{T}$$

where 

ln W_f_ = the natural logarithm of the final weight.

ln W_i_ = the natural logarithm of the initial weight.

T = time (days) between lnW_f_ and ln W_i._

$$\mathrm{Survival}\;\mathrm{rate}\;\%\;\left(\mathrm{SR}\right)=\frac{\mathrm{Number}\;\mathrm{of}\;\mathrm{fish}\;\mathrm{at}\;\mathrm{the}\;\mathrm{End}\;\mathrm{of}\;\mathrm{the}\;\mathrm{Experiment}}{\mathrm{Number}\;\mathrm{of}\;\mathrm{fish}\;\mathrm{at}\;\mathrm{the}\;\mathrm{Beginning}\;\mathrm{of}\;\mathrm{the}\;\mathrm{Experiment}}\times 100$$

$$\mathrm{Feed}\;\mathrm{Conversion}\;\mathrm{Ratio}\;\left(\mathrm{FCR}\right)=\frac{\mathrm{Dry}\;\mathrm{feed}\;\mathrm{intake}\;\left(g\right)}{\mathrm{Fish}\;\mathrm{live}\;\mathrm{weight}\;\mathrm{gain}\;\left(g\right)}$$

$$\mathrm{Condition}\;\mathrm{factor}\;\left(\mathrm{CF}\right)=\frac{\left(\mathrm{Final}\;\mathrm{weight}\right)}{{(\mathrm{Final}\;\mathrm{length}}^{3})}\times 100$$

#### 2.8.5. Statistical Analysis

All the experiments were conducted in triplicate. The data is presented as mean ± standard deviation. To analyze the data, one-way analysis of variance (ANOVA) was used. The *p* value ≤ 0.05 was considered statistically significant. All of the statistical analysis was conducted using SPSS software version 22.

## 3. Results

### 3.1. Isolation of Bacteria and Their Antimicrobial Activity

A total of 60 bacterial strains were isolated from the gastrointestinal tracts of *Channa Punctatus* and *Channa striatus* in the summer season (April 28 °C) of year 2021. Out of 60 strains, only 15 were Gram positive, as shown in Table 1.

Among them, only two VIZ: PBG9D and BCS10 (1), exhibited antagonistic activity against the Gram-negative bacteria *Aeromonas hydrophila*; however, none of the strains showed antagonistic activity against *Pseudomonas aeruginosa*. PBG9D was isolated from *Channa punctatus*, while BCS10 (1) was isolated from *Channa Striatus*. Antagonistic activity was determined by the zone of inhibition at the intersection points of the isolated strain and the test pathogen. Table 2 sheds light on the antimicrobial activity of the bacterial strains.

### 3.2. pH and Bile Tolerance

**Figure 3:**
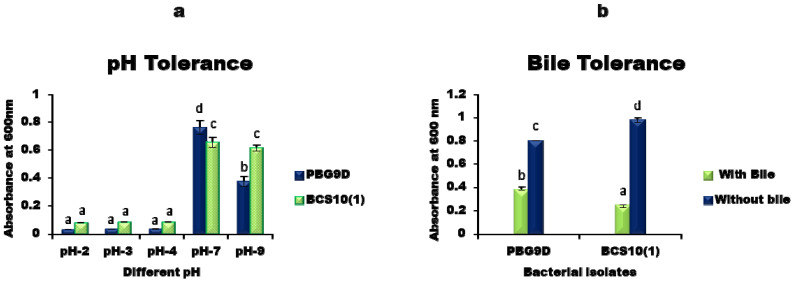
(**a**) Bacterial growth at different pH. (**b**) Bile tolerance potential of isolated bacterial strains. Values represent mean ± SD (n = 3). Different letters on bars indicate significant difference (*p* ≤ 0.05) between groups (PBG9D and BCS10 (1)).

The selected bacterial isolates produced satisfactory results in pH tolerance tests under different culture conditions. Both isolates showed significantly similar growth within the pH range of 2–4. At pH 7, the isolate (PBG9D) showed the highest growth rate compared to that of the isolate (BSC10 (1)). However, at pH 9, PBG9D showed a decrease in growth and BCS10 (1) showed significantly similar growth to that of pH 7. This finding shows that both the isolates could survive in acidic as well as in alkaline conditions, but nonetheless, flourishing growth was demonstrated at neutral pH 7. In the bile tolerance test, both isolates exhibited significant growth in the presence and absence of bile salt (0.3% ox gall). However, PBG9D isolated from *Channa Punctatus* exhibited the highest tolerance to bile salt compared to that of BCS10 (1) (Figure 3a,b).

### 3.3. Antibiotic Susceptibility

The results of antibiotic susceptibility tests of the selected bacterial strains are shown in Table 3. Both isolates were highly susceptible to ampicillin, kanamycin, neomycin, erythromycin, and tetracycline (greater than 10 mm zone of inhibition) and were found to be resistant to two antibiotics, including amoxicillin and penicillin.

### 3.4. Adhesion and Biofilm Formation

**Figure 4:**
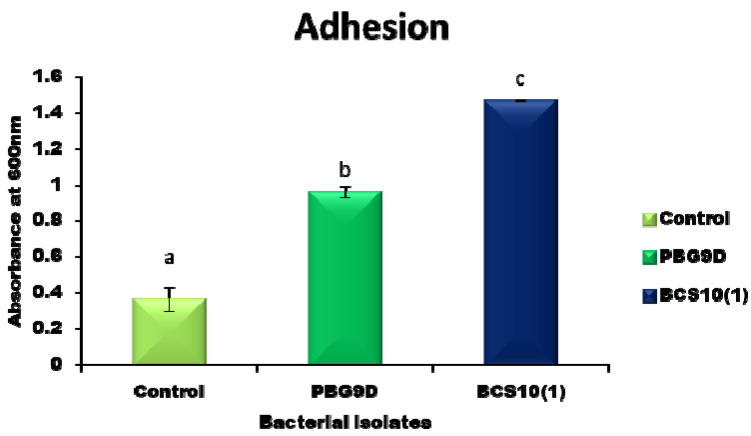
In vitro adhesion capacity of bacterial isolates. Values represent mean ± SD (n = 3). Different letters on bars indicate significant difference (*p* ≤ 0.05) between groups (PBG9D and BCS10 (1)).

For selection of a potential probiotic, the adhesion property of a bacteria is an important criterion. Both isolates showed better adhesion capacity than the control (Figure 4). Among these, the isolate BCS10(1) showed the highest (*p* ≤ 0.05) adhesion capacity followed by PBG9D.

### 3.5. Identification of Isolates

On the basis of biochemical characterization, the strains PBG9D and BCS10 (1) were found to be rod-shaped and Gram-positive. It was also found that both bacterial isolates could utilize glucose and catalase. All other compounds, such as urease, gelatin, and indole, were not utilized by both strains. The results of biochemical characterization showed similarity to that of *Bacillus* sp. (Table 4).

**Figure 5:**
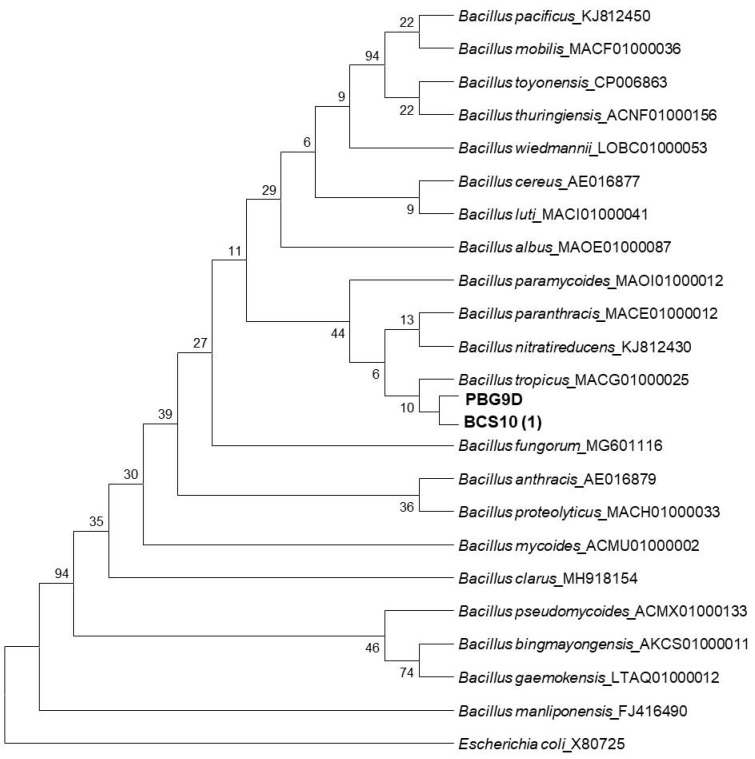
Phylogeny tree showing closely related species of the isolates.

For molecular characterization, 16s ribosomal RNA gene amplification was conducted at the Microbial Type Culture Collection (MTCC), Chandigarh, Punjab, India. Both strains showed high sequence homology with *Bacillus paramycoides* (100%). Evolutionary analysis was conducted in MEGA7 (Figure 5).

### 3.6. Growth Performance

**Figure 6:**
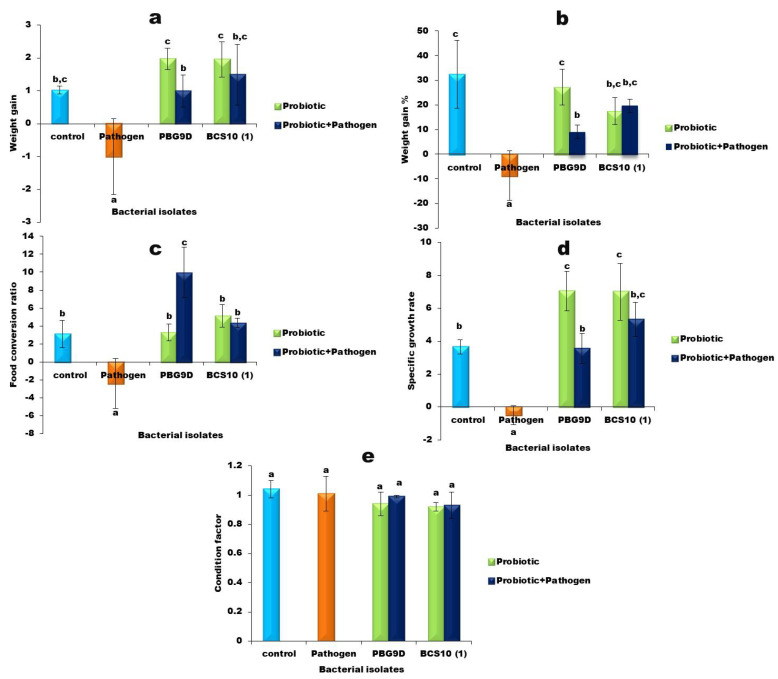
Growth parameters of *Labeo rohita* fed with basal and experimental diets for 28 days. (**a**) Weight gain, (**b**) weight gain %, (**c**) feed conversion ratio, (**d**) specific growth rate, (**e**) condition factor. Values represent mean ± SD. Different letters on bars indicate significant difference (*p* ≤ 0.05) between groups (PBG9D and BCS10 (1)).

The growth performance of *Labeo rohita* fed with basal and experimental diets is presented in Figure 6. There were no significant differences in weight gain and weight gain % between fish in the control (I), probiotic (III) and (IV), and probiotic with pathogen (V) and (VI) groups. However, fish in the pathogen (II) group exhibited significantly lower weight gain and weight gain % compared to all other groups. The FCR of fish in the control (I), probiotic (III) and (IV), and probiotic with pathogen (V) and (VI) groups were significantly similar, except for PBG9D and *Aeromonas hydrophila*. The FCR of fish in the pathogen (II) group was significantly different from the FCR of fish in other groups. Survival rate (SR) and condition factor (CF) were not significantly different among all groups. No mortality was observed in any group. The specific growth rate (SGR) was higher in fish in the probiotic (III) and (IV) groups compared to other groups. The SGR of fish in the control (I) and probiotic with pathogen (V) and (VI) groups were not significantly different; however, a lower SGR was exhibited by the pathogen (II) group.

## 4. Discussion

There is increasing evidence that *Bacillus* sp. can effectively inhibit the growth of fish pathogens belonging to genera *Aeromonas* sp., *Streptococcus* sp., *Staphylococcus* sp., and *Vibrio* sp., [44,45]. The antagonistic activity of *Bacillus* strains is attributed to the production of various inhibitory substances, including bacteriocins, siderophores, hydrogen peroxide, bacitracin, and so on [45,46,47]. In this study, the isolates PBG9D and BCS10 (1) considerably inhibited the growth of fish pathogenic bacteria, namely *Aeromonas hydrophila*. Similarly, Ramesh [25] examined the antagonistic activity of *Bacillus* isolates against different *Aeromonas* sp. and reported that the isolates exhibited inhibition properties. For effective colonization and metabolic activity in the intestinal tract, potential probiotic bacteria must be resistant to low pH and bile salts [25]. In the present investigation, PBG9D and BCS10 (1) isolates showed resistance to acidic and alkaline pH; however, higher survivability was observed at pH 7. Our results suggest that the isolates can survive and grow in the intestinal bulb of *Labeo rohita* as the pH of intestinal fluid ranges from 6.8–7.1 [48]. The isolates also showed tolerance to 0.3% bile salts, which has been reported to be a critical concentration for the selection of bile-resistant strains [32].

Apart from antagonistic activity and resistance to the intestinal environment, both isolates PBG9D and BCS10 (1) were found highly susceptible to the majority of tested antibiotics, including ampicillin, kanamycin, neomycin, erythromycin, and tetracycline, but were resistant to amoxicillin and penicillin. Similar results were reported by Govindaraj [26] who has found susceptibility of lactic acid bacteria to the tested antibiotics, including vancomycin, ciprofloxacin, norfloxacin, and imipenem, but resistance to ampicillin, amoxicillin, penicillin, and gentamicin antibiotics. Furthermore, Thankappan et al [33] have reported the susceptibility of *Bacillus* spp. to the tested antibiotics, such as ampicillin, tetracycline, kanamycin, erythromycin, amoxicillin, penicillin, and gentamicin. The intrinsic resistance mechanism of probiotic bacteria may help to maintain beneficial microbiota in the intestine after antibiotic treatments [26,49].

Colonization among the intestinal epithelial cells is an important trait of probiotic bacteria in order to occupy all of the intestine’s accessible space and prevent the adhesion of pathogenic bacteria [31,50]. Furthermore, adhesion to the intestinal epithelial cells enhances the potential of probiotics to stimulate the immune system [29,51]. Adhesion is a process that involves interaction of the bacterial cell membrane with the glycocalyx, a layer composed of glycolipids and glycoproteins that protects intestinal epithelial cells from mechanical damage. This layer also contains mucin sugar residues that act as a ligand for bacterial membrane receptors. Additionally, it is thought that adhesion is facilitated by a number of passive forces, including hydrophobic interaction, electrostatic interaction, and steric interaction [29,52]. In the present study, both isolates exhibited good adhesion capacity, fulfilling the important criteria of probiotic selection. Adhesion capacity of *Bacillus* isolates was also reported by [31,38].

Different studies have reported that probiotics have the ability to control diseases in cultured systems through various modes of action [53]. It has also been reported that probiotics, when administered through diet, improve weight gain, specific growth rate, feed conversion ratio, survival rate, condition factor, and immune responses in the host [36,54,55]. In the present study, we observed no significant difference in weight gain, weight gain %, or food conversion ratio in the probiotic (III) and (IV) groups compared to the control. However, the specific growth rate in the probiotic (III) and (IV) groups was significantly increased compared to other groups. This finding was in accordance with the results of previous research [56,57]. In spite of having no significant difference in weight gain of fish in the control (I) and probiotic (III) and (IV) groups, a numerically high WG was observed in the fish groups fed with PBG9D and BCS10 (1)-enriched diet, which affirms the effect of probiotics. Furthermore, in the probiotic with pathogen (V) and (VI) groups, weight gain, weight gain %, food conversion ratio, and specific growth rate were significantly increased compared to the pathogen (II) group, confirming the probiotic effect of producing inhibitory substances, as mentioned earlier [45,58]. In the present study, survival rate among all groups was not significantly different. The results are in accordance with the previous research reported by [59]. However, abnormal swimming and loss of appetite are considered clinical symptoms of *Aeromonas* infection [60]. The pathogen (II) group exhibited similar signs and weight loss but not mortality.

Condition factor is considered as an important parameter for understanding the well-being of cultured fish. To quantify the well-being of fish, fishery biologists use the length and weight relationship provided by an index known as condition factor ‘K’. High K value of fish represents heavy for their length, and vice versa. In this study, no significant difference was observed in the condition factor of rohu fingerlings among all groups. Our results are in agreement with those of Chaudhary [61], who reported that the condition factor of *Labeo rohita* was near to a value of 1. Furthermore, Shadarack [56] observed similar results while examining the condition factor of juvenile amberjack. Thus, the overall results of our study clearly reflect that both strains of *Bacillus paramycoides* could enhance specific growth rate and disease resistance in *Labeo rohita*. To the best of our knowledge, the present study is the first to reveal that *Bacillus paramycoides* strains isolated from *Channa punctatus* and *Channa striatus* can be used as potential probiotics to inhibit pathogen growth.

## 5. Conclusions

In conclusion, under in vitro conditions, both strains of *Bacillus paramycoides* showed better probiotic properties, such as antimicrobial activity, tolerance to pH and bile salts and in vitro adhesion properties. Furthermore, in vivo evaluation provides evidence that dietary supplementation with *Bacillus paramycoides* (PBG9D and BCS10 (1)) at 10^8^ CFU/g for 4 weeks can enhance specific growth rate and disease resistance against *Aeromonas hydrophila* in *Labeo rohita*. However, further studies should be conducted on other fish pathogens’ haematological and immune responses to fully demonstrate their mode of action in fish.

## Figures and Tables

**Table 1:** Total bacterial strains isolated from *Channa punctatus* and *Channa striatus*.

Total Isolated Bacterial Strains		
*Channa punctatus*	*Channa striatus*	*Gram* positive
PBG1, PBG2, PBG3, PBG4, PBG5, PBG6, PBG7A, PBG7B, PBG7C, PBG7D, PBG7E, PBG8, PBG9A, PBG9B, PBG9C, PBG9D, PBG9E, PBG10	BCS1 (1), BCS1 (2), BCS3, BCS4, BCS5, BCS6, BCS7, BCS8, BCS9, BCS10 (1), BCS10 (2), BCS11, BCS12, BCS13, BCS14, BCS15, BCS16, BCS17, BCS18 (1), BSS18 (2), BCS19, BCS20 (1), BCS20 (2), MCS1, MCS2, MCS3, MCS4, MCS5, MCS6, MCS7, MCS8, MCS9, MCS10, MCS11, MCS12, MCS13, MCS14, MCS15, MCS16, MCS17, MCS18, MCS19	PBG9D, BCS1 (1), BCS1 (2), BCS10 (1), BCS10 (2), BCS18 (1), BCS19, BCS20 (1), BCS20 (2), MCS6, MCS15, PBG5, PBG1, BCS4, BCS6

**Table 2:** Antimicrobial activity of bacterial strains against fish pathogens.

S. No	Bacterial Strains	*Aeromonas hydrophila*	*Pseudomonas aeruginosa*
01	PBG9D	+++	−
02	BCS10(1)	++	−

The +++ symbol represents complete inhibition, with >10 mm zone of inhibition; ++ symbol represents moderate inhibition, with between 5–10 mm zone of inhibition; and the − symbol represents no inhibition.

**Table 3:** Antibiotic susceptibility of selected bacterial isolates.

S. No.	Antibiotics	PBG9D	BCS10 (1)
01	Ampicillin (25 µg)	S	S
02	Neomycin (30 µg)	S	S
03	Amoxicillin (10 µg)	R	R
04	Erythromycin (15 µg)	S	S
05	Tetracycline (30 µg)	S	S
06	Kanamycin (30 µg)	S	S
07	Penicillin (10U)	R	R

S represents susceptibility, R represents resistance.

**Table 4:** Biochemical characterization of bacterial strains isolated from fish gut.

Biochemical Tests	PBG9D	BCS10 (1)
Gram staining	+	+
Motility test	−	−
Catalase test	+	+
Urease test	−	−
Indole test	−	−
Gelatin test	−	−
Glucose fermentation	+	+
Lactose fermentation	−	−
Methyl red	+	+
Voges–Proskauer	−	−

+ sign represents positive reaction, − sign represents negative reaction.

## Data Availability

The molecular sequence 16S rRNA of *Bacillus paramycoides* (PBG9D and BCS10 (1)) is available at Genbank, NCBI, with Accession numbers, OQ300496 and OQ303631. The data generated or analyzed during the study are available on reasonable request. All the data are present in this article.
